# Enhanced bioremediation of triclocarban-contaminated soil by *Rhodococcus rhodochrous* BX2 and *Pseudomonas* sp. LY-1 immobilized on biochar and microbial community response

**DOI:** 10.3389/fmicb.2023.1168902

**Published:** 2023-03-30

**Authors:** Lei Miao, Siyuan Chen, Hua Yang, Yaqi Hong, Liwen Sun, Jie Yang, Guanjun Sun, Yi Liu, Chunyan Li, Hailian Zang, Yi Cheng

**Affiliations:** ^1^College of Resources and Environment, Northeast Agricultural University, Harbin, China; ^2^Key Laboratory of Swine Facilities Engineering, Ministry of Agriculture and Rural Affairs, Harbin, China; ^3^College of Plant Protection, Northeast Agricultural University, Harbin, China

**Keywords:** triclocarban, biodegradation, immobilization, intermediates, bacterial community

## Abstract

Triclocarban (TCC), an emerging organic contaminant (EOC), has become a severe threat to soil microbial communities and ecological security. Here, the TCC-degrading strain *Rhodococcus rhodochrous* BX2 and DCA-degrading strain *Pseudomonas* sp. LY-1 (together referred to as TC1) were immobilized on biochar to remove TCC and its intermediates in TCC-contaminated soil. High-throughput sequencing was used to investigate the microbial community structure in TCC-contaminated soil. Analysis of co-occurrence networks was used to explore the mutual relationships among soil microbiome members. The results showed that the immobilized TC1 significantly increased the removal efficiency of TCC from 84.7 to 92.7% compared to CK (no TC1 cells on biochar) in 10 mg/L TCC liquid medium. The utilization of immobilized TC1 also significantly accelerated the removal of TCC from contaminated soil. Microbial community analysis revealed the crucial microorganisms and their functional enzymes participating in TCC degradation in soil. Moreover, the internal labor division patterns and connections of TCC-degrading microbes, with a focus on strains BX2 and LY-1, were unraveled by co-occurrence networks analysis. This work provides a promising strategy to facilitate the bioremediation of TCC in soil, which has potential application value for sustainable biobased economies.

## 1. Introduction

Triclocarban (TCC) is a typical broad-spectrum antibacterial agent that is extensively utilized in cosmetics, pharmaceuticals and personal care products (PPCPs) because of its strong bactericidal efficacy (Wang et al., 2018; Yang et al., 2021b). The widespread production and consumption of TCC have turned it into one of the most universally detected pollutants in natural surroundings and it has been graded as an emerging organic contaminant (EOC; Yun et al., 2020; Li et al., 2022). In the United States, an approximated 2.75 × 10^5^ kg of TCC has been found annually in wastewater treatment plants (WWTPs; Carey et al., 2016), which is not completely removed during the WWT process and is released into agricultural soils *via* irrigation with wastewater effluents and biosolid applications (Cha and Cupples, 2010; Blair et al., 2015). It has been reported that the TCC concentrations in sewage sludge-amended soil reached 2,715 μg/kg in the United States and 4,940–8,000 μg/kg in Canada (Edwards et al., 2009; Gottschall et al., 2012). In China, the application of biosolids led to 1,584 μg/kg of TCC being transferred into soils (Yun et al., 2020). Furthermore, evidence on the uptake of TCC by crops and the transfer of TCC to the food chain through agricultural soil has been presented (Thelusmond et al., 2019). In addition, long-term exposure to TCC can negatively affect soil nutrients, ammonification, and nitrification and, to some extent, leads to structural changes in the soil indigenous microbiome (Snyder et al., 2011; Carey et al., 2016). Therefore, it is necessary to develop suitable and low-cost approaches to eliminate TCC effectively and efficiently from the contaminated environment.

Compared with chemical and physical remediation methods, biodegradation is considered an efficient, low-energy-consuming, and pollution-free method to remove TCC from the environment (Wei et al., 2022; Wang et al., 2022a; An et al., 2023a). However, given the recalcitrant and antimicrobial characteristics of TCC, bacteria that can effectively degrade TCC in soil are limited (Halden and Paull, 2005; Tarnow et al., 2013). To date, only *Ochrobactrum* sp. TCC-2 and *Diaphorobacter* sp. LD72 have been reported to degrade TCC in contaminated soil (Liang et al., 2020). The removal efficiency of 5 mg/kg TCC by coculture of TCC-degrading strain TCC-2 and LD72 reached 93.0% within 30 days, which indicated that inoculated functional degraders and indigenous microorganisms worked together to degrade TCC and its intermediates (Liang et al., 2020). Nevertheless, the coculture of strain TCC-2 and LD72 required a relatively long time to degrade TCC. Furthermore, the crucial microorganisms and their functional enzymes associated with the TCC degradation process in TCC-contaminated soil have not been fully revealed.

In this context, a combination of adsorption and biodegradation can significantly enhance TCC removal efficiency and shorten TCC degradation time in the environment. Jenjaiwit et al. (2021) investigated the TCC removal from wastewater treatment using immobilized microorganisms only during a batch experiment, which illustrated that the TCC removal ability of immobilized microorganisms was better than biochar (no microorganisms). However, the bioremediation of TCC-contaminated soil by immobilized functional microorganisms and its influences on the composition and function of the native soil microbiome have not been well studied.

The main reported TCC transformation pathway is hydrolysis to form 3,4-dichloroaniline (3,4-DCA) and 4-chloroaniline (4-CA; Sipahutar and Vangnai, 2017; Yun et al., 2017; Li et al., 2022). However, the oxidation of these two TCC intermediates may additionally be the rate-limiting step during TCC degradation. In our previous study, we enriched the TCC-degrading bacterium *Rhodococcus rhodochrous* BX2 from activated sludge, which exhibits high efficiency in TCC degradation (Li et al., 2022). In addition, *Pseudomonas* sp. LY-1 was also found to have a high degradation ability of 3,4-DCA (unpublished). The coculture of strain BX2 and strain LY-1 increased the TCC removal efficiency of strain BX2 from 76.8 to 89.7%.

Therefore, *R. rhodochrous* BX2 and Pseudomonas sp. LY-1 (together referred to as TC1) were selected for the bioremediation of TCC-contaminated soil by immobilization on biochar. This study was carried out to (1) determine the optimal conditions for the removal of TCC by biochar and immobilized TC1; (2) examine the removal efficiency of TCC and its intermediates by biochar and immobilized TC1 in TCC-contaminated soil; (3) explore the shift in microbial community structure before and after the remediation of TCC-contaminated soil with biochar and immobilized TC1; (4) reveal the crucial microorganisms and their functional enzymes participating in TCC degradation in TCC-contaminated soil; and (5) investigate the internal labor division patterns and connections of TCC-degrading strains in the microbial community of TCC-contaminated soil under immobilized TC1 treatment. This work demonstrates the potential of immobilized microorganisms to enhance TCC removal from soils, paving the way for targeted bioremediation strategies.

## 2. Materials and methods

### 2.1. Strains, chemicals, and culture media

The bacterial coculture system TC1 for TCC degradation comprised *R. rhodochrous* BX2 and *Pseudomonas* sp. LY-1. Strain BX2 and strain LY-1 were isolated from activated sludge of Jiangsu Hormone Research Institute and Heilongjiang Pesticide Factory Institute, respectively (Li et al., 2013). Triclocarban (TCC, ≥98%) and intermediates, including 3,4-dichloroaniline (3,4-DCA, 98%) and 4-chloroaniline (4-CA, 99%), were procured from Aladdin (Shanghai, China) for chemical analysis. Acetonitrile, acetone, methanol, and other chemicals employed for analysis and experiment were all high-performance liquid chromatography (HPLC)-grade and acquired from Fisher Scientific (Shanghai, China). Strain BX2 and LY-1 cultivation involved enrichment and culturing using LB medium and modified mineral salt medium (MMSM) optimized in our previous study (Li et al., 2022).

### 2.2. Cornstalk, biochar, and soil samples

The cornstalk that was used for preparing biochar and test soils was taken from Northeast Agriculture University’s experimental farm (Harbin, China). Pyrolysis of the prepared biochar was performed under a nitrogen atmosphere at 300, 500, and 700°C for 2 h in a tube furnace (SX-G07123, Zhonghuan Electric Furnace Co., Ltd., Shanghai, China) as stated by Ma et al. (2021), which were labeled BC_300_, BC_500_, and BC_700_, respectively. The test soils were homogenized and sieved using a 2 mm mesh. The soil physicochemical characteristics are as follows: 38.6 g/kg soil organic matter (SOM), 1.25 g/kg total nitrogen (TN), 0.790 g/kg total phosphorus (TP), 214 mg/kg total potassium (TK), 51.1 mg/kg available phosphorus (AP) and 46.1 mg/kg available potassium (AK), water-holding capacity 5.01%, and pH 7.06.

### 2.3. Adsorption capability of TCC by biochars

To ascertain the TCC adsorption capability and equilibrium time of biochar, a preliminary TCC adsorption kinetic experiment was performed. Biochar sample (20 mg) was added to 10 mg/L TCC MMSM (50 mL) and cultured at a rotary shaken at 160 rpm for 48 h at 25°C. The residual TCC concentrations was examined by 1 mL continuous sampling. The adsorption capability of biochar was carried out by applying equation (1). The pseudo–first–order and pseudo–second–order models were performed to estimate TCC adsorption kinetics, as shown in equations (2)–(5) (Ho and McKay, 1999).


(1) $$q=q_{0}e^{kt}$$

(2) $$q=-kt+q_{0}$$

(3) $$q=\frac{1}{x}\frac{\text{dS}}{\text{dt}}$$

(4) $$q=\frac{q_{\max}S}{K_{s}+S+\frac{S^{2}}{K_{i}}}$$

(5) $$V=\frac{V_{\max}}{1+2\sqrt{k_{s}/k_{i}}}$$

Based on single-factor experimental data, a central composite design (CCD) was utilized to further increase the TCC adsorption ability of biochar. According to the critical parameters of the TCC adsorption process their interactions, rotation speed (A), addition amount (B), and TCC concentration (C) were preferred as factors, and the TCC removal efficiency was the unique response variable. The experimental design was made up of 20 runs, and each factor and its value are presented in Supplementary Table S1. The residual TCC concentration was ascertained by HPLC, and the HPLC detection conditions of TCC are detailed in section 2.5. Supplementary Table S1 listed the coding levels of TCC removal experimental parameters and independent variables. The response surface was described as an empirical model portrayed by equation (6) (Miao et al., 2023) as follows:


(6) $$Y=A+\sum_{j=1}^{k}A_{j}X_{j}+\sum_{j=1}^{k}A_{\text{jj}}X_{j}^{2}+\underset{i}{\sum }\sum_{<j=2}^{k}A_{\text{ij}}X_{i}X_{j}$$

In the equation, Y represents the response value, X refers to the input variable, and A, A*j*, A*jj*, and A*ij* denote the intercept, linear, quadratic, and interaction coefficients, respectively.

### 2.4. Preparation and optimization conditions of immobilized TC1 for TCC degradation

The cultivation and immobilization processes of strains BX2 and LY-1 were performed according to the method of Jenjaiwit et al. (2021). The immobilized system containing strain BX2 and strain LY-1 was named immobilized TC1. The biochar was characterized by specific surface area and pore size using an automatic volumetric sorption analyzer (Autosorb 1 MP, Quantachrome, United States), and results were obtained using the Brunauer–Emmett–Teller (BET) equation. Scanning electron microscopy (SEM; SU8010, HITACHI; Japan) was used to observe the surfaces of immobilized TC1. CCD was carried out to further improve the removal efficiency of TCC by immobilized TC1 (Supplementary Table S2). Addition amount (A), TCC concentrations (B), and temperature (C) were the factors, and the TCC removal efficiency was the response variable. Statistical analysis and surface response analysis methods were the same as in section 2.3.

### 2.5. Biodegradation of TCC and its metabolites by immobilized TC1

To compare the TCC removal efficiency of immobilized microorganisms and biochar, 10 mg/L TCC + 3% biochar (L-TB) and 10 mg/L TCC + 3% immobilized TC1 (L-TCIB) treatment groups were set up, and the concentrations of TCC and its intermediates were measured by HPLC. TCC, 3,4-DCA, and 4-CA in the MMSM were extracted by dichloromethane as depicted by Li et al. (2022). All samples were filtered *via* 0.22-μm disposable organic filter membranes and their concentrations of TCC and intermediates were measured through HPLC (model-2695, Waters Co., Milford, MA, United States) with a 4.6 mm × 250 mm column (C18, Agilent). The mobile phase was water and acetonitrile (40:60; v/v). An ultraviolet detector monitoring at 275 nm (Yun et al., 2017).

### 2.6. Bioremediation of TCC-contaminated soil by immobilized TC1

After mixing TCC with acetone at a specific ratio, it was added to 30 g of air-dried soil and stirred while dripping to facilitate smooth mixing. The soil was left for a while and after acetone was fully volatilized, 30 g TCC-contaminated soil and 270 g air-dried soil were mixed evenly. Water was added every 24 h to insure that the soil moisture content was 25%. The soil experiment consisted of one control group and six treatment groups (S-CK: 10 mg/kg TCC-contaminated soil; S-TB: 10 mg/kg TCC-contaminated soil + 3% biochar; S-BX2: 10 mg/kg TCC-contaminated+3% biochar + free strain BX2; S-LY-1: 10 mg/kg TCC-contaminated + 3% biochar + free strain LY-1; S-TBC: 10 mg/kg TCC-contaminated soil + 3% biochar + free strain BX2 + free strain LY-1; and S-TCIB: 10 mg/kg TCC soil + 3% immobilized TC1). Samples were taken at 0, 4, 8, 12, 16, and 20 days. The extraction method of TCC and its metabolites from soil was described by Liang et al. (2020), and the TCC residues in the soil were determined by HPLC.

### 2.7. Soil basic properties and enzyme analysis

The soil pH was determined by the approach of Gao et al. (2019). The determination methods of SOM, TN, TP, TK, AP, and AK were performed by soil agrochemical analysis by Bao (2008). The polyphenol oxidase activity in soil was determined by pyrogallol colorimetry following the manner of Chen et al. (2016). Dehydrogenase activity in soil was determined by a spectrophotometer (Chaperon and Sauvé, 2008; Campos et al., 2019). Soil urease activity was examined by the phenol-sodium hypochlorite colorimetric method (Chaperon and Sauvé, 2008). The activities of soil β-glucosidase and N-acetyl-R-aminoglycosides were measured using kits.

### 2.8. Analysis of soil bacterial communities by 16S rRNA sequencing

Based on the results of the soil degradation experiments, S-CK, S-TB, and S-TCIB samples at 20 days were selected for sequencing. High-throughput sequencing of the V3-V4 region of the 16S rRNA gene was carried out by Sangon Biotech Co., Ltd. (Shanghai, China) to analyze the function and structure of bacterial communities in soil samples (Cheng et al., 2022; Xu et al., 2023).

### 2.9. Statistical analysis

All experiments, including those for the control group, were carried out in triplicate. One-way ANOVA was used to compare data between different groups with a significance level of *p* < 0.05 or *p* < 0.001 (Zhao et al., 2022). The relevant data in this study were analyzed by SPSS software 22.0 (Jiang et al., 2022; Li et al., 2023). The Mantel test was performed with R4.2.1. Network analysis was conducted with Gephi 0.9.7 according to Spearman’s correlation coefficients (*p* < 0.01; An et al., 2023b).

## 3. Results and discussion

### 3.1. Determination of biochar adsorption capacity and optimization of adsorption conditions for TCC

**Figure 1:**
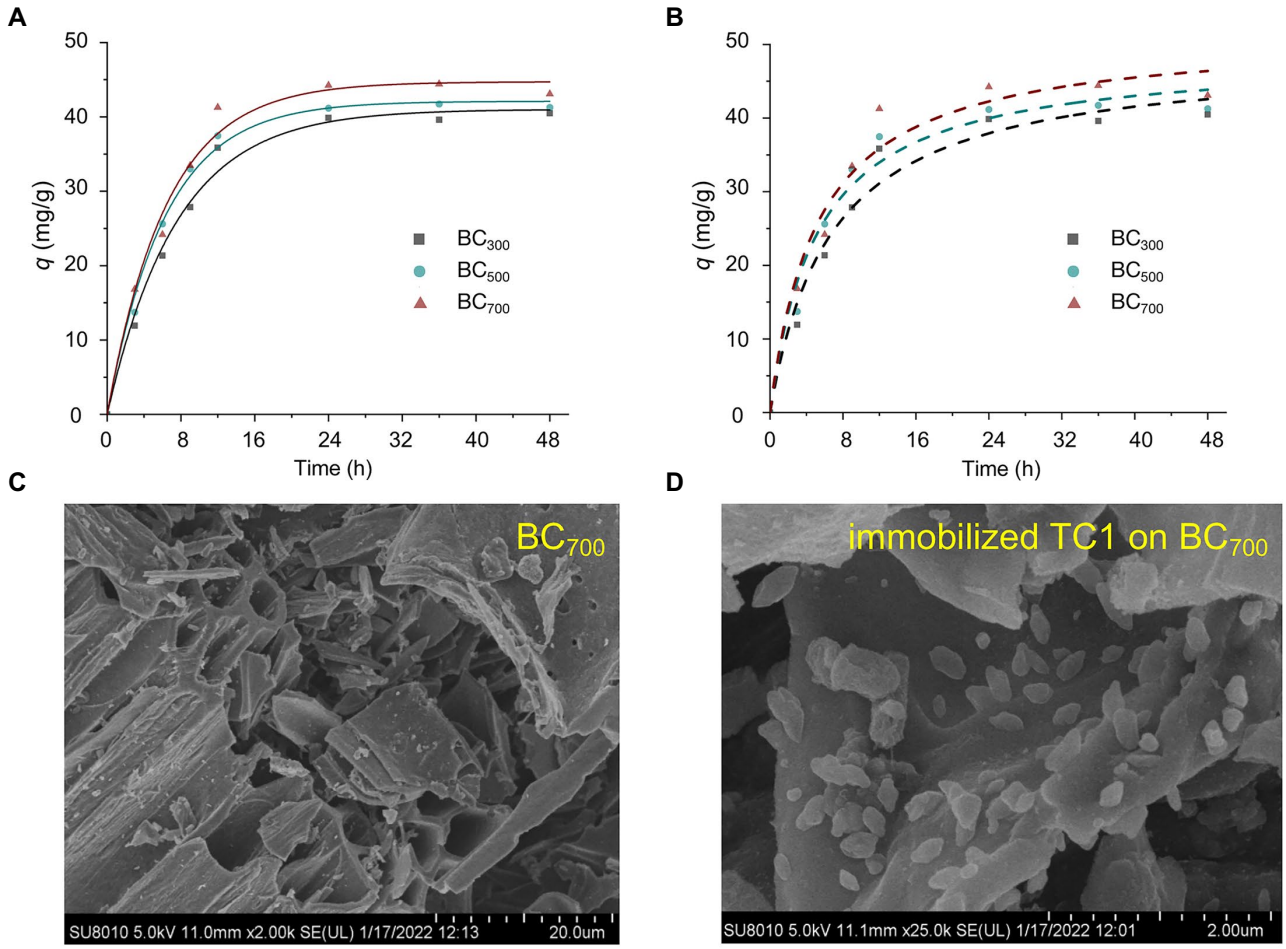
Fitting curves of the pseudo-first order **(A)** and pseudo-second order **(B)** adsorption kinetic models of BC_300_, BC_500_, and BC_700_. SEM images of biochar before **(C)** and after the immobilization of TC1 **(D)**.

Biochars prepared at 300, 500, and 700°C, named BC_300_, BC_500_, and BC_700_, respectively, were used for the TCC adsorption kinetics study. The pseudo-first order and pseudo-second order kinetic models were implemented to TCC adsorption experimental data (Figures 1A,B). At an initial TCC concentration of 10 mg/L, TCC was rapidly adsorbed onto the biochar and reached a adsorption plateau 12–24 h later. The final TCC removal efficiency of BC_300_, BC_500_, and BC_700_ were 71.2, 74.3, and 79.8%, respectively. TCC has low water solubility and high octanol–water partitioning coefficient, which leads to rapid and efficient adsorption of TCC by biochar (Halden and Paull, 2005).

Moreover, the pseudo-first order models (*R*^2^ = 0.987–0.994) fitted the data lightly greater than pseudo-second order models (*R*^2^ = 0.967–0.973) for the adsorption of TCC, which implied that the pore diffusion process was the rate-limiting step (Supplementary Table S3; Alberti et al., 2012). It was also confirmed that all adsorption rate constants (*k_1_*) were much better than k_2_ (Supplementary Table S3; Qi et al., 2022). Combined with the results of SEM and BET, BC_700_ increased the surface area, pore volume, and pore diameter of biochar compared with BC_300_ and BC_500_, which facilitated the diffusion of TCC in the pores of biochar, thus increasing the TCC adsorption efficiency of biochar (Supplementary Figure S1).

To further explore the ability of biochars to adsorb TCC at higher concentrations, the pseudo-first order kinetic model was used to simulate the adsorption effects of BC_700_ at TCC concentrations of 30 and 50 mg/L. The consequences indicated that the adsorption efficiency reached 51.5 and 79.8 mg/g, respectively, suggesting that biochar can also be used as an effective adsorbent for high concentrations of TCC. According to the adsorption kinetics and characterization of biochar mentioned above, BC_700_ provided the strongest TCC adsorption capability, so it was applied in the subsequent experiments.

To increase the removal efficiency of TCC by biochar (BC_700_), CCD was used to optimize the preparation and adsorption conditions of biochar according to the single-factor test results (Supplementary Figures S2A–C). The regression coefficient *R*^2^_Pred_ is 0.9822, and *R*^2^_Adj_ is 0.9763, which suggests the accuracy of the model (*R*^2^ > 0.9). CCD analysis was performed on three factors at three levels. The TCC removal efficiency by biochar ranged from 74.3 to 84.9%. Based on multivariate regression analysis of experimental data, the second-order polynomial equation was calculated as follows:


$$\begin{array}{l} Y=8.778+3.42X_{1}+1.52X_{2}+0.19{\mathrm{6X}}_{3}+\\ \;0.32{\mathrm{3X}}_{1}{\;}^{2}+0.43X_{2}{\;}^{2}\_0.31X_{3}{\;}^{2}\_\\ \;1.56X_{1}X_{2}\_2.57X_{1}X_{3}+0.55{\mathrm{9X}}_{2}X_{3} \end{array}$$

where Y denotes the TCC removal efficiency; X_1_, X_2_, and X_3_ represent independent factors (rotation speed, addition amount, and TCC concentration); X_1_X_2_, X_1_X_3_, and X_2_X_3_ are interaction factors; and X_1_^2^, X_2_^2^, and X_3_^2^ are quadratic items.

The *p* value of X_1_X_2_ < 0.0001 proved that the interaction between rotation speed and addition amount was highly significant (Jiang et al., 2020). The *p* value of X_1_X_3_ < 0.05 indicated that the rotation speed and TCC concentration had a significant impact on the TCC removal efficiency (Zhao et al., 2023). Contour lines were selected to assess the influences of X_1_X_2_ and X_1_X_3_ interactions on the TCC removal efficiency (Supplementary Figures S2D,E). When each variable was at the middle level, the maximal removal efficiency was indicated by the contour lines in the central area of each contour line and the extreme value that was located in this region. Analysis of the regression equation and contour lines suggested that the optimal adsorption conditions for biochar were as follows: rotation speed, 160 rpm; addition amount, 3%; and TCC concentration, 10 mg/L. Under optimal cultivation conditions, the removal efficiency of TCC by biochar reached 84.7%.

### 3.2. Optimization of TCC degradation conditions with the addition of immobilized TC1

Based on the results of our previous experiments, a 5 ml bacterial suspension loading and 9 h immobilization time were selected to prepare immobilized TC1 for subsequent studies (data not shown). The SEM image of immobilized TC1 showed that the cells successfully adhere to the biochar surface (Figures 1C,D). CCD was executed to further improve the removal efficiency of TCC according to the single-factor test results (Supplementary Figures S3A–C). The *R*^2^_Pred_ (0.9860) and *R*^2^_Adj_ (0.9808) of CCD pointed out the accuracy of model (Wang et al., 2022b). The TCC removal efficiency obtained by immobilized TC1 was 73.8–94.5%. The second-order polynomial equation was as follows:


Y=9.218–0.75X1–0.105X2  +1.44X3+0.281X1 2+0.694X2 2  –0.932X3 2–1.94X1X2  +2.50X1X3–0.948X2X3

where Y represents the TCC removal efficiency; X_1_, X_2_, and X_3_ denote independent factors (addition amount, TCC concentration, and temperature); X_1_X_2_, X_1_X_3_, and X_2_X_3_ are interaction factors; and X_1_^2^, X_2_^2^, and X_3_^2^ are quadratic items.

Contour lines were chosen to assess the effects of the interactions between the independent factors X_1_X_2_ (*p* < 0.0001) and X_1_X_3_ (*p* < 0.05) on the TCC removal efficiency at an intermediate level (Supplementary Figures S3D,E). These analyses implied that the optimal cultivation conditions for immobilized TC1 were an addition amount of 3%, TCC concentration of 10 mg/L, and temperature of 25°C. Under optimal cultivation conditions, the degradation efficiency of TCC reached 92.7%.

### 3.3. Biodegradation of TCC and its intermediates by immobilized TC1

**Figure 2:**
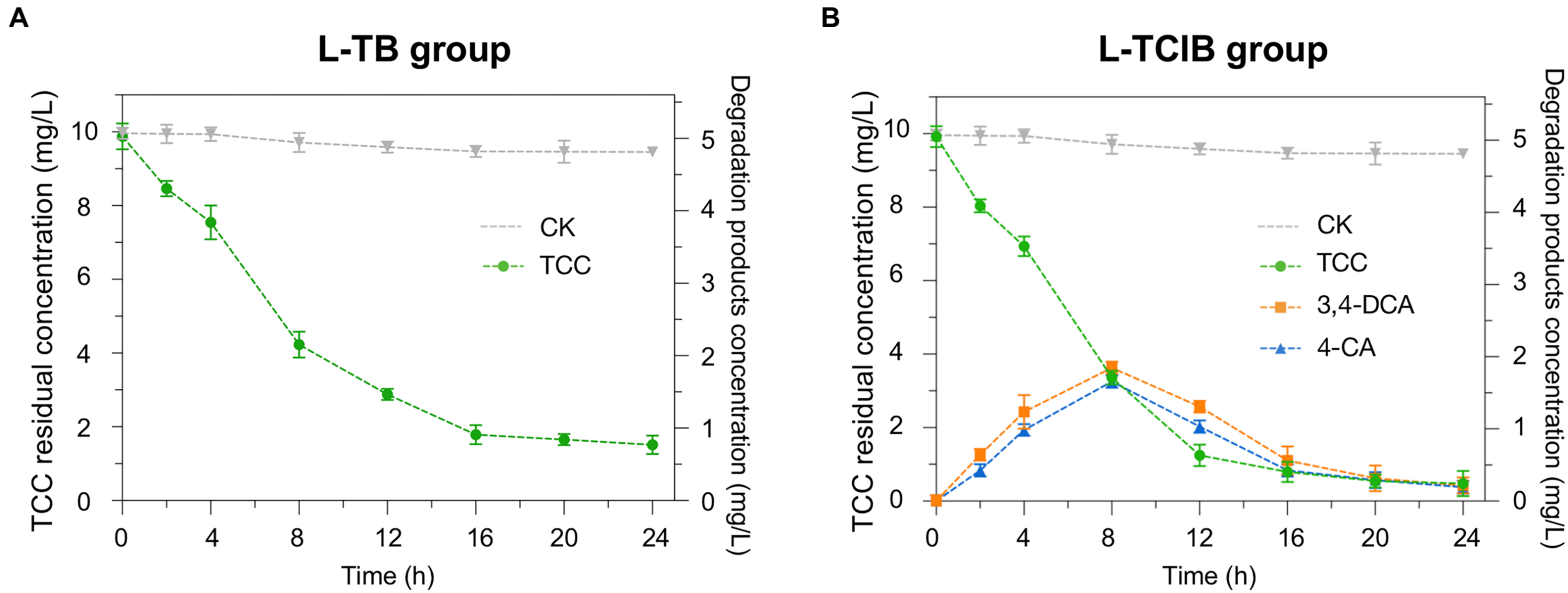
The removal efficiency of TCC and its intermediates by biochar treatment **(A)** and immobilized TC1 treatment **(B)**.

Based on our previous research and relevant literature, the primary degradation step of TCC is hydrolysis of the amide bonds, yielding 3,4-DCA and 4-CA (Mulla et al., 2016; Sipahutar and Vangnai, 2017; Yun et al., 2017; Li et al., 2022). TCC, 3,4-DCA and 4-CA have been reported to be toxic to humans and other organisms; therefore, the removal efficiency of biochar (L-TB) and immobilized TC1 (L-TCIB) for the above products was examined. In the L-TB group, the concentration of TCC was 1.78 mg/L at 24 h, which corresponded to a removal efficiency of 84.7% (Figure 2A). In the L-TCIB group, the concentration of TCC reached a minimum value of 0.476 mg/L at 24 h, corresponding to a removal efficiency of 92.7% (Figure 2B). The results revealed that immobilized TC1 was more effective than biochar in removing TCC.

For the removal efficiency of TCC degradation products in the L-TB group, 3,4-DCA and 4-CA were not found, suggesting that the removal of TCC by biochar was only through adsorption. In L-TCIB, 3,4-DCA and 4-CA increased to the maximum concentrations (3.63 and 3.25 mg/L) at 8 h and then gradually decreased from 8 to 24 h (0.432 and 0.381 mg/L), indicating that the TCC removal by immobilized TC1 was through adsorption by biochar and biodegradation of microorganisms, i.e., the loose and porous biochar was first used as an adsorbent to adsorb TCC from the aqueous medium into the pores of biochar. Then, it was degraded by the immobilized strains BX2 and LY-1 to the less toxic products, which were further degraded to small molecular compounds through metabolic division of labor.

Taweetanawanit et al. (2019) reported that the removal efficiency of 10 mg/L TCC by sodium alginate-immobilized *Pseudomonas fluorescens* MC46 was 72.0%. In this study, immobilized TC1 was able to achieve 92.7% removal efficiency of TCC, possessing a more robust TCC degradation capability. This also demonstrated that biochar is an excellent carrier for cell immobilization, probably because biochar reduces the contact of microbial cells with toxic compounds, resulting in greater removal performance by immobilized microorganisms (Jenjaiwit et al., 2021). These results imply that immobilized TC1 might be a potential a notable candidate for biodegradation in TCC-contaminated environments.

### 3.4. Bioremediation of TCC-contaminated soil

**Figure 3:**
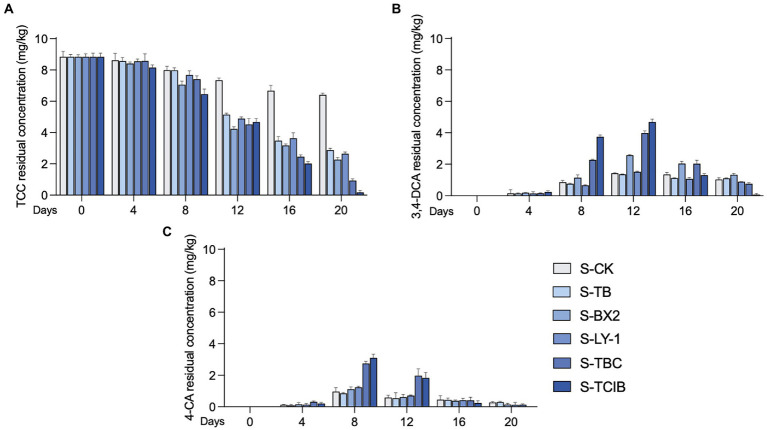
The removal efficiency of TCC **(A)** and its intermediates 3,4-DCA **(B)** and 4-CA **(C)** in TCC-contaminated soil. S-CK: 10 mg/kg TCC-contaminated soil; S-TB: 10 mg/kg TCC-contaminated soil + 3% biochar; S-BX2: 10 mg/kg TCC-contaminated + 3% biochar + free strain BX2; S-LY-1: 10 mg/kg TCC-contaminated + 3% biochar + free strain LY-1; S-TBC: 10 mg/kg TCC-contaminated soil + 3% biochar + free strain BX2 + free strain LY-1; and S-TCIB: 10 mg/kg TCC soil + 3% immobilized TC1.

The TCC removal efficiency by immobilized TC1 was further examined in TCC-contaminated soil (10 mg/kg; Figure 3). This concentration was estimated based on the accumulation of TCC in sewage sludge-amended soils ranging from 1.58 to 8 mg/kg (Edwards et al., 2009; Liang et al., 2020). Based on the results of biodegradation of TCC and its intermediates in liquid medium and the confirmation of chemical standards. The degradation of TCC in soil still involves the transformation of 3,4-DCA and 4-*CA*. Hence, the residual concentrations of TCC, 3,4-DCA and 4-CA were mainly examined in the bioremediation tests of TCC-contaminated soil. For the S-CK group, only 27.5% of TCC was observed to be degraded at 20 days, with a residual concentration of 6.41 mg/kg. In addition, 1.43 mg/kg 3,4-DCA accumulated at 12 days, and 0.965 mg/kg 4-CA accumulated at 8 days, which could not be completely degraded at 20 days (residual concentrations of 3,4-DCA and 4-CA: 1.03 and 0.273 mg/kg). This suggests that some native microorganisms in the soil present the ability to degrade TCC, but the self-cleaning capability of the soil was restricted. In the S-TB group, the residual concentration of TCC at 20 days was 2.88 mg/kg, corresponding to a removal efficiency of 67.5%, which indicated that the biochar has a certain adsorption capacity, but loses its ability to affect TCC once it reaches adsorption equilibrium so that TCC cannot be well removed. In addition, small concentrations of 3,4-DCA and 4-CA were also detected in the S-TB group, probably because of the degradation of TCC by native microorganisms. For the S-BX2 group, with the degradation of TCC, 3,4-DCA and 4-CA accumulated up to 2.57 mg/kg at 12 days and 1.13 mg/kg at 8 days, respectively, which suggests that the degradation of TCC is a result of a combination of the adsorption of biochar and the degradation of immobilized strain BX2. At 20 days, the residual concentration of TCC was 2.27 mg/kg, and the accumulated 4-CA was further degraded with a residual concentration of 0.840 mg/kg, while 3,4-DCA could not be further degraded with a residual concentration of 1.53 mg/kg. This result was consistent with our previous findings that strain BX2 degraded TCC to 3,4-DCA and 4-CA and continued to degrade 4-CA but not 3,4-DCA (Li et al., 2022). For the S-LY-1 group, the decrease in TCC residual concentration (2.64 mg/kg at 20 days) was mainly due to adsorption by biochar, as strain LY-1 was unable to degrade TCC. For the S-TBC group, with the addition of free biochar, strain BX2 and strain LY-1 to TCC-contaminated soil, TCC was continuously degraded and 3.99 mg/kg 3,4-DCA accumulated at 12 days and 2.75 mg/kg 4-CA accumulated at 8 days. At 20 days, the residue concentrations of TCC, 3,4-DCA and 4-CA decreased to 1.23, 0.764, and 0.792 mg/kg, respectively, showing that biochar adsorption and the added functional microorganisms worked together to degrade TCC and its intermediates. For the S-TCIB group, the residue concentration of TCC was further decreased to 0.773 mg/kg at 20 days compared with the other groups, and only a small amount of accumulated 3,4-DCA and 4-CA (0.072 and 0.0123 mg/kg, respectively) was detected. This was because biochar not only played the role of adsorption but also provided suitable colonization sites and reaction sites for strains BX2 and LY-1 by immobilization, making immobilized TC1 more conducive for microorganisms to convert TCC into other less toxic products. The above results indicated that immobilized TC1 was an excellent candidate for the practical biodegradation of TCC in contaminated environments.

### 3.5. Responses of the microbial community to TCC degradation

To further explore the reason that immobilized TC1 promotes the degradation of TCC, the effect of S-TCIB on the soil bacteria community was calculated (S-CK and S-TB served as controls). A total of 412,384 high-quality bacterial V3–V4 Illumina sequences, ranging from 38,753 sequences to 54,907 sequences per sample, were acquired for further analysis.

#### 3.5.1. Alpha diversity of the microbial community

**Figure 4:**
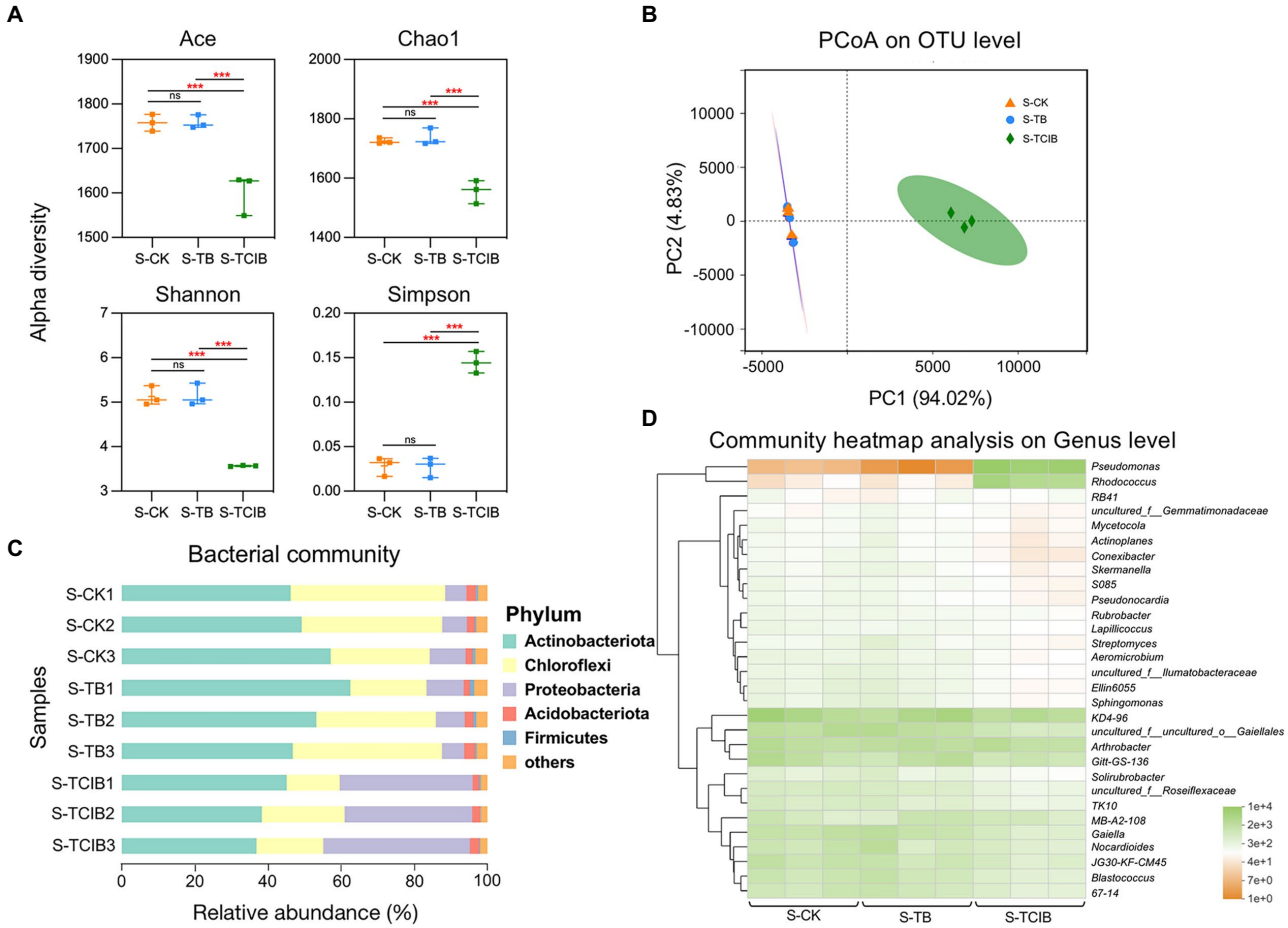
Differences in alpha diversity among S-CK, S-TB, and S-TCIB **(A)**. PCoA analysis among S-CK, S-TB, and S-TCIB **(B)**. Bar plot showing bacterial taxonomic composition at the phylum level in S-CK, S-TB, and S-TCIB **(C)**. Heatmap showing the top 30 bacterial taxonomic compositions at the genus level in S-CK, S-TB, and S-TCIB **(D)**. ANOVA was used to compare differences among groups. S-CK: 10 mg/kg TCC-contaminated soil; S-TB: 10 mg/kg TCC-contaminated soil + 3% biochar; S-TCIB: 10 mg/kg TCC soil + 3% immobilized TC1.

As shown in Figure 4A, no significant difference in alpha diversity indices between the S-CK and S-TB groups, which corresponded with the research of Cole et al. (2019), indicating the overall stability of different bacteria communities in biochar-amended soils. In contrast, the species richness (ACE and Chao1 indices) and soil bacterial diversity (Shannon and Simpson indices) were meaningfully lower in S-TCIB than in S-CK and S-TB (*p* < 0.05). These discoveries approximate to those of Li et al. (2021), in which bioaugmentation of *Bacillus subtilis* TTL1 led to decreased species richness and bacterial diversity in soil. The inoculation of exogenous microorganism strains BX2 and LY-1 resulted in competition for nutrients and changed the habitat of indigenous microbes, therefore reducing the alpha diversity of soil. In addition, PCoA showed that the treatment group (S-TCIB) was separated from the control groups (S-CK and S-TB; Adonis *R*^2^ = 0.9376, *p* < 0.05; Figure 4B). The samples from S-CK and S-TB were clustered but separated from those of S-TCIB. As indicated by these results, immobilized TC1 reduced the bacterial community diversity in TCC-contaminated soil.

#### 3.5.2. Structures of the microbial community

The impacts of different treatment groups on the bacterial community structures were investigated at the phylum and genus levels (Figure 4C). At the phylum level, bacterial species with a relative abundance of >1% were screened for cluster analysis, and the phylum with a relative abundance of >10% was classified as the dominant phylum. S-CK was dominated by Actinobacteria (53.2%) and Chloroflexi (36.1%), accounting for 89.3% of the total bacterial diversity. S-TB was also dominated by Actinobacteria (56.9%) and Chloroflexi (31.7%), accounting for 88.6% of the total bacterial diversity. The relative abundances of Actinobacteria and Chloroflexi remained stable in the S-CK and S-TB groups and were in general the dominant phyla in polluted soils (detection rate over 70%), which was connected with the degradation of high molecular-weight organic matter, including TCC (Miura et al., 2007). In the S-TCIB group, with the addition of immobilized TC1, the relative abundance of Actinobacteria (41.5%) and Chloroflexi (18.8%) decreased, while the relative abundance of Proteobacteria (37.0%) increased to become the dominant phylum. Proteobacteria has often been reported to be enriched in agricultural soil related to the biodegradation of polycyclic aromatic hydrocarbons (PAHs), diclofenac, carbamazepine and TCC (Yu et al., 2017; Thelusmond et al., 2019). Proteobacteria was also the dominant phylum in waste-activated sludge supplemented with TCC (Wang et al., 2017). In this study, the added immobilized strains BX2 and LY-1 belonged to Actinobacteria and Proteobacteria, respectively. However, due to the limited carbon and nutrient sources in the soil, competition for resources among the bacteria occurred, and the relative abundance of Proteobacteria increased, leading to a corresponding decrease in the abundance of other phyla. The relative abundance of Actinobacteria decreased slightly but remained the dominant phylum in the TCC-contaminated soil.

At the genus level, the top 30 bacterial genera were screened for cluster analysis, and the genus with a relative abundance of >1% was classified as the dominant genus (Figure 4D). After the addition of biochar and immobilized TC1, the relative abundance of the dominant genera *KD4-96*, *Gitt-GS-136*, *JG30-KF-CM45*, *TK10* and *Solirubrobacter* decreased from 15.8, 7.09, 4.92, 2.21, and 1.34% in S-CK to 14.1, 6.65, 3.82, 1.82, and 1.30% in S-TB to 9.22, 3.91, 1.89, and 0.913% in S-TCIB, respectively (Figure 4D). The genera KD4-96, Gitt-GS-136, and *Solirubrobacter* have been reported to be related to soil nutrient cycling (Hemmat-Jou et al., 2018; Yang et al., 2021a). The genera *JG30-KF-CM45* and *TK10* play key roles in removing organic compounds from soil (Yang et al., 2021a; Yuan et al., 2022). These genera were most abundant in the S-CK group, recommending that they may play an essential role in removing TCC from untreated TCC-contaminated soil. In the S-TB group, adding biochar increased the relative abundance of the genera *Gaiella*, *Nocardioides*, *Blastococcus*, *MB-A2-108, 67-14*, *Ellin6055*, *Sphingomonas*, and *Streptomyces* in the soil. These bacteria have revealed the capacity to degrade some refractory organics or emerging contaminants (Ali et al., 2017; Du et al., 2022; Ya et al., 2022). Therefore, in the S-TB group, TCC removal was mainly accomplished through a combination of adsorption of TCC by biochar and biodegradation of TCC by the bacteria mentioned above.

*Pseudomonas* sp. significantly increased from 0.024% in S-CK and 0.005% in S-TB to 33.7% in S-TCIB. *Rhodococcus* sp. also increased from 0.204% in S-CK and 0.206% in S-TB to 13.7% in S-TCIB. The relative abundance of other dominant genera did not increase significantly in S-TCIB. Reported microbial species that can degrade TCC effectively are confined, considering the recalcitrant and antimicrobial features of TCC (Tarnow et al., 2013). To date, the genera discovered to degrade TCC only include *Sphingomonas* sp. (Mulla et al., 2016), *Pseudomonas* sp. (Taweetanawanit et al., 2019), *Ochrobactrum* sp. (Sipahutar and Vangnai, 2017; Yun et al., 2017), and *Rhodococcus* sp. (Li et al., 2022). Among them, *Sphingomonas* sp. was found in the S-TCIB treatment group; however, the relative abundance of *Sphingomonas* sp. was lower in S-TCIB (0.724%) than in S-CK (1.99%) and S-TB (2.09%). Moreover, *Ochrobactrum* sp. was not detected. Hence, *Rhodococcus* sp. and *Pseudomonas* sp. might be the major contributors to the bioremediation of TCC-contaminated soil in the S-TCIB group.

#### 3.5.3. Effects of TCC on predicted metabolic pathways and functional enzymes

**Figure 5:**
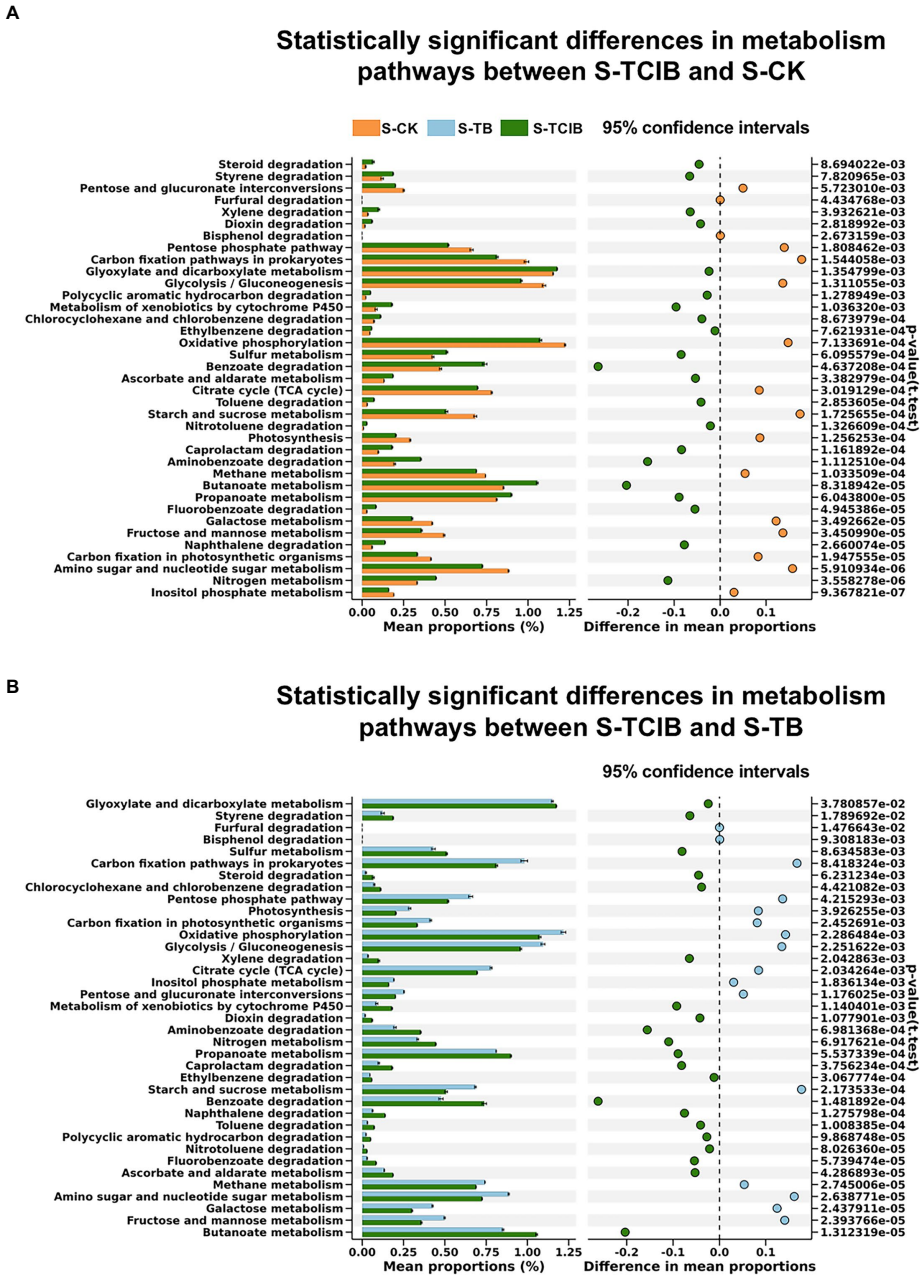
Statistically significant differences in the metabolic pathways associated with TCC degradation among the samples of S-TCIB and S-CK **(A)** and S-TCIB and S-TB **(B)**. The points to the left of the dashed line indicate greater values in the sample microcosms compared to the controls. Conversely, those to the right show the opposite trend. S-CK: 10 mg/kg TCC-contaminated soil; S-TB: 10 mg/kg TCC-contaminated soil + 3% biochar; S-TCIB: 10 mg/kg TCC soil + 3% immobilized TC1.

The PICRUSt enrichment analysis that was based on the KEGG databases was executed to supply more information on bacteria functional gene expression comprehensively. It has been reported that the pathway annotated as “xenobiotic biodegradation and metabolism” is closely related to the microbial degradation of TCC and its intermediates (Li et al., 2022). Therefore, a three-level pathway analysis of the “xenobiotic biodegradation and metabolism” of the samples in the three treatments was conducted. As shown in Figure 5, the number of metabolic pathways increased by 21 in S-TCIB compared to both S-CK and S-TB. These pathways contain a large number of functional genes related to hydroxylation deamination, ring cleavage, and oxidation of TCC, which can further metabolize TCC into small molecule compounds.

**Figure 6:**
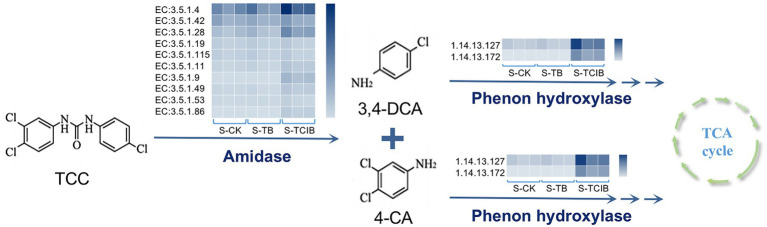
The abundance of functional genes related to TCC degradation predicted using PICRUSt analysis in the S-CK, S-TB, and S-TCIB groups. S-CK: 10 mg/kg TCC-contaminated soil; S-TB: 10 mg/kg TCC-contaminated soil + 3% biochar; S-TCIB: 10 mg/kg TCC soil + 3% immobilized TC1.

Based on the degradation products confirmed by chemical standards in soil bioremediation experiments and related literature, the main intermediates of TCC degradation were 3,4-DCA and 4-CA (Mulla et al., 2016; Sipahutar and Vangnai, 2017; Yun et al., 2017). As depicted in Figure 6, the abundance of predicted amidase [EC:3.5.1.-] in S-TCIB was higher than that in S-TB and S-CK, which has been proven to be responsible for converting TCC to 3,4-DCA and 4-CA (Li et al., 2022). Subsequently, intermediates 3,4-DCA and 4-CA were hydroxylated by putative phenol hydroxylase (Li et al., 2022). The abundance of putative phenol hydroxylase [EC:1.14.13.-] in S-TCIB was also higher than that in S-TB and S-CK, suggesting that the added exogenous degrading bacteria BX2 and LY-1 might promote the expression of these enzymes and thus enhance the transformation of TCC in soil.

#### 3.5.4. Microbial interaction networks in TCC-degrading microbiomes

**Figure 7:**
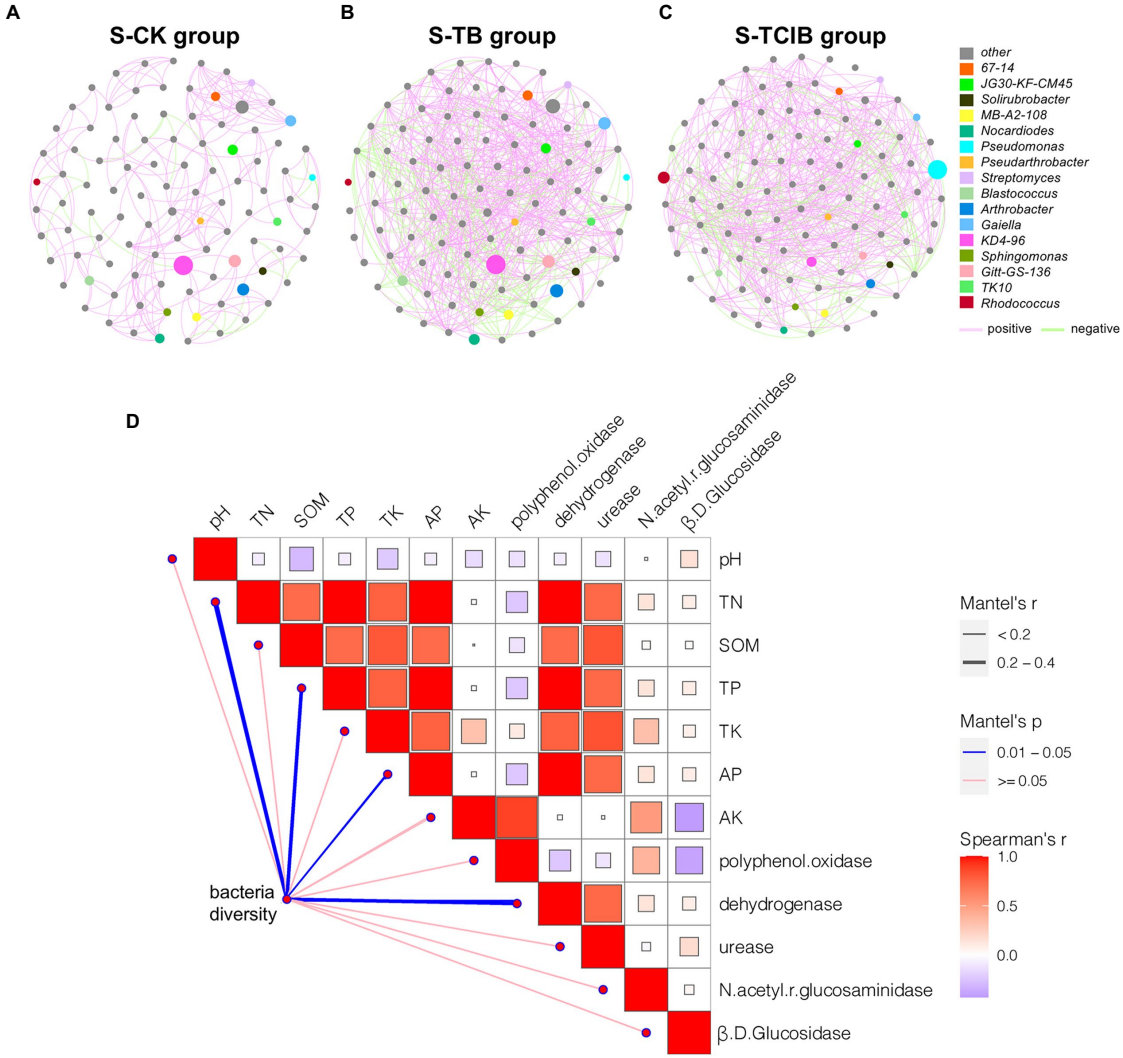
Network analysis showing the co-occurrence patterns for the bacteria (top 100 genera) in S-CK **(A)**, S-TB **(B)**, and S-TCIB **(C)** based on Spearman’s correlation coefficients (*p* < 0.01). The red and green links represent positive and negative interactions, respectively. The node sizes were proportional to the node degrees (the number of connections). **D**: Mantel test results showing the correlations between bacterial diversity and multiple indicators. S-CK: 10 mg/kg TCC-contaminated soil; S-TB: 10 mg/kg TCC-contaminated soil +3% biochar; S-TCIB: 10 mg/kg TCC soil +3% immobilized TC1.

The mutual relationships between soil microbiome members (OTUs) can be reflected by co-occurrence networks based on correlation coefficient matrices to a certain extent. Therefore, to better understand the impacts of immobilized TC1 on the potential interrelationships between native bacterial community members, the co-occurrence networks of S-CK, S-TB and S-TCIB were constructed (Figures 7A–C). The network analysis showed that the complexity of the S-TCIB network (79.7% positive interactions) was higher than that of S-CK (75.5% positive interactions) and S-TB (77.7% positive interactions), indicating that immobilized TC1 positively influenced the interactions among microbial populations. Analysis of key functional bacteria for TCC degradation showed that *Rhodococcus* sp. and *Sphingomonas* sp. included species capable of degrading TCC in S-CK and S-TB, and these two genera showed positive interactions, which indicated that these two genera might cooperate to resist TCC stress. In S-TCIB, the positive interactions between *Rhodococcus* sp. and *Sphingomonas* sp. were almost eliminated, probably because the dominant genera in S-TCIB were changed. In addition, 14.5% of nodes belonged to *Rhodococcus* sp. and 34.3% of nodes belonged to *Pseudomonas* sp. in S-TCIB. *Pseudomonas* became the dominant genus in the S-TCIB network and showed a positive relationship with *Rhodococcus* sp. This is consistent with our previous findings that strains BX2 and LY-1 metabolize TCC and its degradation products through synergistic interactions.

It is expected that soil environmental conditions are also essential in driving bacterial diversity. Mantel test analysis showed that three indices of microbial nutrition contents, TN, TP, and AP, were the common vital factors affecting bacterial diversity with a meaningful correlation (*p* < 0.05; Figure 7D). In fact, some nutrients contained in soil could be considered nutrients for bacteria because most are essential for microbial growth (Tian et al., 2021). Therefore, they may influence soil bacterial diversity by affecting bacterial biomass. Bacterial diversity was also significantly correlated with dehydrogenase (*p* < 0.05; Figure 7D). Dehydrogenase is involved in various physiological and biochemical processes in bacteria; therefore, it was a characterization index of the oxidation capacity of bacteria in soil.

## 4. Conclusion

In this research, a green and sustainable technology was used to remove TCC from soil. The TCC-degrading strain *Rhodococcus rhodochrous* BX2 and DCA-degrading strain *Pseudomonas* sp. LY-1 (together referred to as TC1) were immobilized on biochar, which showed an effective degradation ability of TCC and its intermediates. The application of immobilized TC1 significantly accelerated the removal of TCC from soil. Analysis of the microbial community revealed that *Rhodococcus* sp. and *Pseudomonas* sp. contributed most to the bioremediation of TCC-contaminated soil. PICRUSt analysis predicted that amidase converts TCC to intermediates in TCC-contaminated soil. Besides, the internal labor division patterns and connections within microbial community demonstrated that strains BX2 and LY-1 play a significant role in the removal of TCC in soil. Although this research has demonstrated that the combined use of biochar and degrading bacteria has great potential for *in situ* bioremediation of TCC-contaminated soil, further studies are important to assess the removal efficiency and adaptability of the immobilized microorganisms under microplot and field conditions.

## Data availability statement

The 16S rRNA gene of R. rhodochrous BX2, accession number JN562728 and Pseudomonas sp. LY-1, accession number CP094353 has been deposited in NCBI. The data supporting High-throughput sequencing are available in SRA under accession number PRJNA938954.

## Author contributions

LM wrote the first draft of the manuscript and conducted data correction. SC wrote the sections of the manuscript and performed formal analysis. HY and YH organized the database. LS and JY performed the statistical analysis. GS and YL helped to modify the graphs. HZ and CL assisted the manuscript checking. YC contributed to conception, revised the manuscript, and provided funding support. All authors contributed to the article and approved the submitted version.

## Funding

This work was supported by the National Natural Science Foundation of China (Nos. 42071268 and U22A20443), and the Technological Project of Heilongjiang Province “the open competition mechanism to select the best candidates” (No. 2022ZXJ05C01-01).

## Conflict of interest

The authors declare that the research was conducted in the absence of any commercial or financial relationships that could be construed as a potential conflict of interest.

## Publisher’s note

All claims expressed in this article are solely those of the authors and do not necessarily represent those of their affiliated organizations, or those of the publisher, the editors and the reviewers. Any product that may be evaluated in this article, or claim that may be made by its manufacturer, is not guaranteed or endorsed by the publisher.
