# Supplementary material for: Enhanced bioremediation of triclocarban-contaminated soil by Rhodococcus rhodochrous BX2 and Pseudomonas sp. LY-1 immobilized on biochar and microbial community response
Source: Front Microbiol. 2023 Mar 30;14:1168902. doi: 10.3389/fmicb.2023.1168902 (PMC10098447; doi:10.3389/fmicb.2023.1168902)
Supplement: Supplementary file 1 [file Data_Sheet_1.pdf]

Supplementary Material

Enhanced bioremediation of triclocarban-contaminated soil by *Rhodococcus rhodochrous* BX2 and *Pseudomonas* sp. LY-1 immobilized on biochar and microbial community response

Lei Miao[1,3,†], Siyuan Chen[1,3,†], Hua Yang[1,3], Yaqi Hong[1,3], Liwen Sun[1,3], Jie Yang[1,3], Guanjun Sun[1,3], Yi Liu[1,3], Chunyan Li[1,3], Hailian Zang[1,3], Yi Cheng[2,3,*].

*** Correspondence:** Yi Cheng: chengyi58918@163.com

1 Supplementary Figures and Tables

1.1 Supplementary Figures

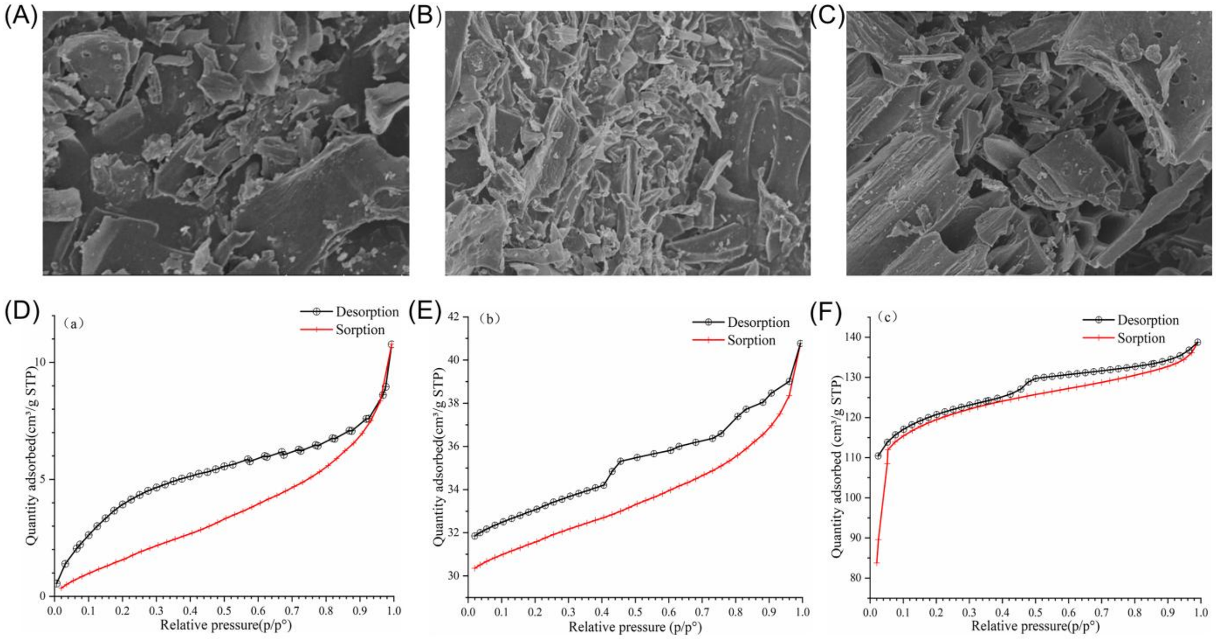

Supplementary Figure 1. Scanning electron micrographs (SEM) of BC_{300} (A), BC_{500} (B), and BC_{700} (C) morphology. N2 adsorption-desorption isotherms of BC_{300} (D), BC_{500} (E), and BC_{700} (F).

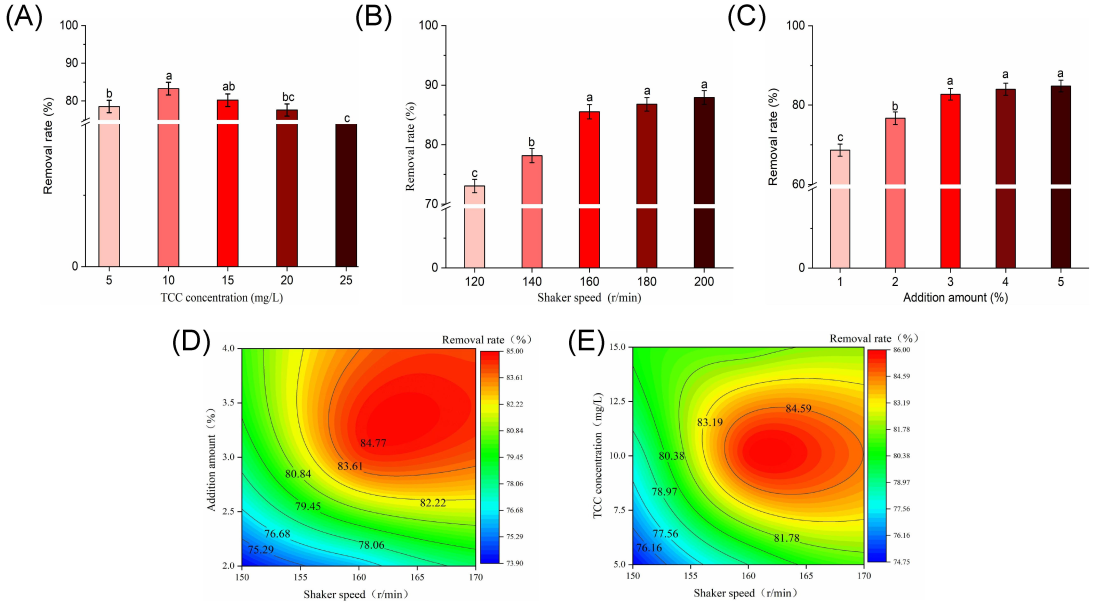

Supplementary Figure 2. Effects of TCC concentration (A), rotation speed (B), and addition amount (C) on the removal efficiency of TCC by biochar. CCD plots showing the effects of various factors and their interactions on the removal efficiency of TCC by biochar (D-E).

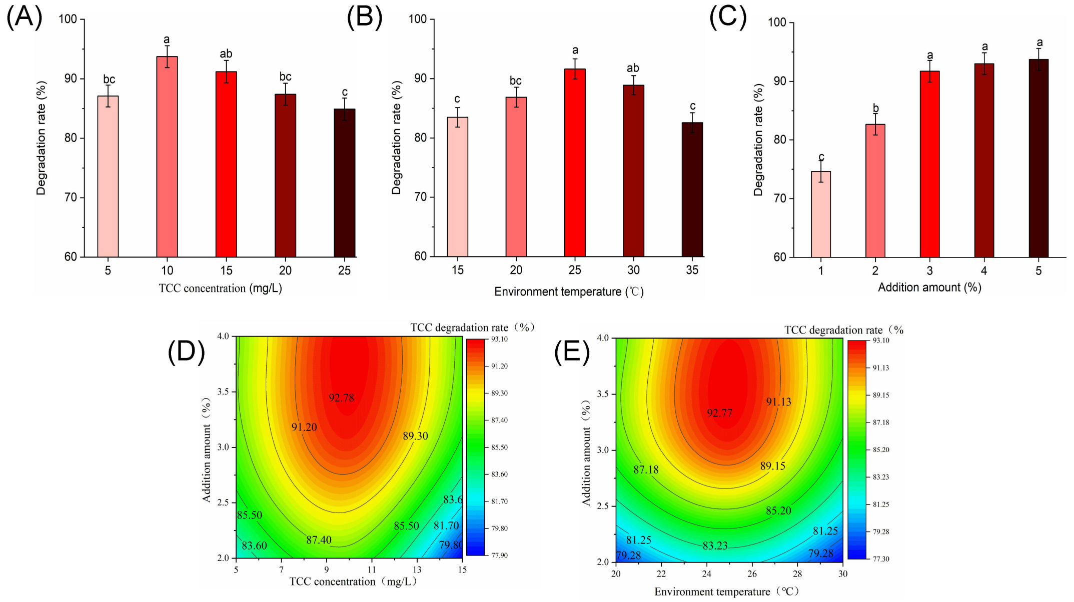

Supplementary Figure 3. Effects of TCC concentration (A), environmental temperature (B), and addition amount (C) on the removal efficiency of TCC by immobilized TC1. CCD plots showing the effects of various factors and their interactions on the removal efficiency of TCC by immobilized TC1 (D-E).

1.2 Supplementary Tables

Supplementary Table 1. Central composite experimental design

	Rotation speed (rpm)	Addition amount (%)	TCC concentration (mg/L)	Removal efficiency of TCC (%) Measured value	Predicted value
1	160	3	5	81.82	81.48
2	170	2	10	78.11	77.95
3	150	4	10	82.28	82.34
4	150	3	-5	82.92	82.77
5	160	3	10	84.86	84.73
6	160	2	15	74.27	74.38
7	170	4	5	83.02	83.31
8	170	3	15	81.99	81.84
9	160	4	15	82.36	81.74
10	150	3	15	79.63	80.52
11	150	2	10	81.05	80.38
12	160	3	5	81.59	79.98
13	170	3	5	77.14	76.14
14	160	2	5	75.96	76.61
15	160	3	10	76.57	77.89
16	150	2	5	75.52	76.79
17	170	3	10	84.04	84.23
18	170	4	10	83.91	83.93
19	160	4	10	83.64	83.78
20	150	4	15	81.13	81.26
Levels of the independent variables in the Central Composite Design					
Low (−1)	150	2	5		
Mid. (0)	160	3	10		
High (1)	170	4	15		

Note: Central composite design (3 factors and 3 levels); R^2_{Pred}=0.9822; R^2_{Adj} =0.9763.

Supplementary Table 2. Central composite experimental design

	Addition amount (%)	TCC concentration (mg/L)	Temperature (℃)	Removal efficiency of TCC (%)	
				Measured value	Predicted value
1	4	10	25	92.88	93.25
2	3	15	25	88.41	89.13
3	2	15	30	72.04	71.79
4	3	10	25	94.49	94.52
5	4	10	20	83.62	83.81
6	2	10	25	73.79	75.10
7	4	5	20	82.92	83.15
8	4	5	30	81.16	80.77
9	4	10	30	82.14	81.47
10	3	5	25	90.28	91.46
11	3	10	20	84.61	87.89
12	2	5	20	78.97	78.84
13	2	15	20	80.20	80.08
14	4	15	20	81.31	81.66
15	4	5	25	80.47	79.14
16	2	5	30	80.81	80.09
17	3	5	20	86.26	85.93
18	2	15	25	83.58	84.65
19	4	15	30	83.88	84.58
20	2	15	25	76.92	77.84
Levels of the independent variables in the Central Composite Design					
Low (−1)	2	5	20		
Mid. (0)	3	10	25		
High (1)	4	15	30		

Note: Central composite design (3 factors and 3 levels); R^2_{Pred}=0.9860; R^2_{Adj} =0.9808.

Supplementary Table 3. Kinetics model fitting parameters of biochar for TCC adsorption

Concentration (mg/L)	Kinetics model	Parameters	Biochars BC300	BC500	BC700
10	Pseudo-first-order	Q_e (mg/g)	7.99	12.28	16.78
		K_1 (h^{-1})	0.13	0.16	0.15
		R^2	0.990	0.994	0.987
	Pseudo-second-order	Q_e (mg/g)	7.86	12.47	16.71
		K_2 (h^{-1})	0.031	0.040	0.037
		R^2	0.972	0.973	0.967
30	Pseudo-first-order	Q_e (mg/g)	8.91	13.25	17.17
		K_1 (h^{-1})	0.23	0.21	0.14
		R^2	0.995	0.983	0.987
50	Pseudo-first-order	Q_e (mg/g)	9.44	14.04	17.92
		K_2 (h^{-1})	0.22	0.25	0.25
		R^2	0.994	0.996	0.991

2 Data Availability Statement

The 16S rRNA gene of *R. rhodochrous* BX2 (accession number JN562728) and *Pseudomonas sp.* LY-1 (accession number CP094353) have been deposited in NCBI. The data supporting High-throughput sequencing are available in SRA under accession number PRJNA938954.
